# Diagnosis and treatment based on quantitative PCR after controlled human malaria infection

**DOI:** 10.1186/s12936-016-1434-z

**Published:** 2016-08-05

**Authors:** Jona Walk, Remko Schats, Marijke C. C. Langenberg, Isaie J. Reuling, Karina Teelen, Meta Roestenberg, Cornelus C. Hermsen, Leo G. Visser, Robert W. Sauerwein

**Affiliations:** 1 Department of Medical Microbiology and Radboud Center for Infectious Diseases, Radboud university medical center, PO Box 9101, 6500 HB Nijmegen, The Netherlands; 2 Department of Infectious Diseases, Leiden University Medical Center, PO Box 9600, 2300 RC Leiden, The Netherlands

**Keywords:** Malaria, *Plasmodium falciparum*, Controlled human malaria infection (CHMI), quantitative PCR (qPCR)

## Abstract

**Background:**

Controlled human malaria infection (CHMI) has become well-established in the evaluation of drugs and vaccines. Anti-malarial treatment is usually initiated when thick blood smears are positive by microscopy. This study explores the effects of using the more sensitive qPCR as the primary diagnostic test.

**Methods:**

1691 diagnostic blood samples were analysed by microscopy and qPCR from 115 volunteers (55 malaria naïve and 60 having received chemoprophylaxis and sporozoite immunization) who were challenged by five mosquitoes infected with *Plasmodium falciparum* sporozoites of the NF54 strain.

**Results:**

Retrospective analysis of different qPCR criteria for diagnosis and treatment, showed that once daily qPCR (threshold 100 parasites/ml) had 99 % sensitivity and 100 % specificity, and shortened the median prepatent period from 10.5 to 7.0 days after CHMI when compared to twice daily measurement of thick blood smears (threshold 4000 parasites/ml). This is expected to result in a 78 % decrease of adverse events before initiation of treatment in future studies. Trial outcome related to infection and protective efficacy remained unchanged.

**Conclusion:**

The use of qPCR as the primary diagnostic test in CHMI decreases symptoms as well as parasitaemia while obviating the need for twice daily follow-up. The implementation improves safety while reducing the clinical burden and costs without compromising the evaluation of protective efficacy.

## Background

Controlled human malaria infection (CHMI) has proven to be a valuable tool to evaluate the efficacy of drugs and vaccines and to study the pathogenesis of clinical malaria. These challenge trials have become highly standardized [1] and are considered a critical step in the clinical development of pre-erythrocytic malaria vaccines [2].

Traditionally, volunteers are followed after CHMI by once to three times daily thick blood smears, and anti-malarial treatment is initiated immediately once two or more parasites are detected by microscopy. In 2004, a standardized protocol for CHMI thick blood smears was introduced using a threshold of 4000 parasites/ml to improve the comparability of study outcomes between centres [3]. Volunteers generally develop submicroscopic parasitaemia for several days before they become thick smear positive. The more sensitive quantitative PCR (qPCR) with a detection limit of 20 parasites/ml was introduced for retrospective analysis feeding a statistical model for more detailed estimation of important parasite parameters including liver load and asexual parasite maturation and multiplication rates [4, 5].

Over the past decade, CHMIs have been performed in over 300 healthy volunteers at Radboud university medical center (Radboudumc), the ‘Harbour Hospital’ in Rotterdam or the Leiden University Medical Centre (LUMC). Despite an acceptable safety profile, CHMIs inevitably cause mild to moderate malaria symptoms such as headache, myalgia and malaise in almost all volunteers, and severe (grade 3) symptoms in about half of volunteers [3, 6]. Moreover, there have been three serious adverse cardiac events shortly after treatment for parasitaemia that have remained incompletely understood [7, 8]. As clinical malaria symptoms are only associated with asexual blood stages, a shorter duration of parasitaemia may reduce the number and severity of adverse events, thereby further minimizing risks and volunteer burden. In addition, treating volunteers before (severe) symptoms occur, would simplify the conduct and follow-up, thereby lowering costs.

In this retrospective study, different thresholds for qPCR diagnostics were analyzed in relation to prepatent period and occurrence of adverse events as well as effects on assessment of protective efficacy.

## Methods

### Study volunteers

Retrospective qPCR data that had previously been generated were collected from nine CHMI trials performed at the Radboud university medical center (Radboudumc), the ‘Harbour Hospital’ in Rotterdam or the Leiden University Medical Centre (LUMC) between 2007 and 2012 [9–15], Table 1.

**Table 1:** Summary of data included in the analysis

	Year	Number of volunteers	CPS-immunization	Patent parasitemia	Pre-patent period^a^		References
					Median	Range	
Study 1	2007	10	3 × 12–15 mosquitoes	0/10	–	–	Roestenberg et al. [13]
		5	–	5/5	9	7–10.5	
Study 2^b^	2007	18	–	18/18	10.5	9–12.5	
Study 3	2009	6	3 × 12–15 mosquitoes^c^	2/6	16.8	15–18.6	Roestenberg et al. [14]
		4	–	4/4	8.5	7.5–10.5	
Study 4	2010	5	–	4/5	10.6	10.6–11	Teirlinck et al. [15]
Study 5	2011	5	3 × 15 mosquitoes	1/5	12	–	Bijker et al. [12]
		9	3 × 10 mosquitoes	1/9	12	–	
		10	3 × 5 mosquitoes	5/10	11	9–15	
		5	–	5/5	9.5	9–13.5	
Study 6	2011	5	3 × 15 mosquitoes	0/5	–	–	Bijker et al. [10]
		5	–	5/5	12.5	9.5–12.5	
Study 7	2012	15	3 × 8 mosquitoes	5/15	12	11–14	Bijker et al. [11]
		4	–	4/4	8.5	7–12	
Study 8	2012	5	–	5/5	10.5	9–10.5	
Study 9	2012	5	–	5/5	12	10.5–16	Bastiaens et al. [9]

Data was included from all malaria naïve or CPS-immunized volunteers undergoing challenge infection with bites from five mosquitoes infected NF54 since 2007

^a^Only volunteers with patent parasitemia included. In all studies pre-patent period is defined as time to positive thick blood smear

^b^Volunteers received three immunization with a candidate malaria vaccine but were unprotected from challenge infection

^c^Rechallenge of CPS-immunized volunteers from Study 1, 2.5 years after immunization and malaria naive controls

All study subjects were healthy female and male volunteers between the age of 18 and 35 years exposed to bites of five *Plasmodium falciparum* NF54 strain infected *Anopheles stephensi* mosquitoes. Prior to challenge infection, 55 volunteers were malaria naïve and 60 had received chemoprophylaxis and sporozoite (CPS) immunization. CPS-immunization was administered via infected mosquito bites at different dosages under chloroquine or mefloquine prophylaxis, as described previously [10–14].

Prior to inclusion, study volunteers were medically screened as described previously [13] and provided written informed consent. All clinical trials were approved by the Radboudumc Committee on Research Involving Human Subjects (CMO) or the Central Committee on Research Involving Human Subjects (CCMO) of the Netherlands.

### Parasitological data

Treatment was initiated after CHMI when a thick blood smear was found positive for parasites. Thick smears were made twice or three times daily and read according to a standard protocol [11]. In short, a slide was considered positive if after reading the number of fields equivalent to 0.5 μL of blood at least two parasites were seen (a threshold of four parasites per µL), and positivity was confirmed by a second independent reader. qPCR assessment was performed according to previously published protocols [16]. qPCR was performed retrospectively from samples taken twice per day from day 5 until day 15 after challenge and once per day from day 16 until day 21.

### Recording of adverse events

Subjects were asked to keep a diary recording symptoms while followed up for adverse events (AEs) on an outpatient basis once or twice daily starting on day 5 after challenge infection until day 21. Adverse events were collected until end of study visits either on day 28 or day 35 after challenge, depending on the study. An adverse event was defined as any undesirable symptom occurring after challenge infection. AEs were defined as grade 1, no interference with daily activity; grade 2, some interference with daily activity; or grade 3, requiring bed rest. The following symptoms were solicited: fever, headache, malaise, fatigue, myalgia, arthalgia, nausea, vomiting, chills, diarrhoea and abdominal pain.

### Statistical analysis

Depending on the study, qPCR data was analysed using Microsoft Excel (version 2007) for Windows or using a specialized electronic Case Report Form program (Hermsen Computer Services) created for Radboudumc CHMI trials. Data was combined using Microsoft Excel 2007 for Windows and statistical analysis was performed using IBM SPSS Statistics 22 for Windows.

## Results

**Fig. 1:**
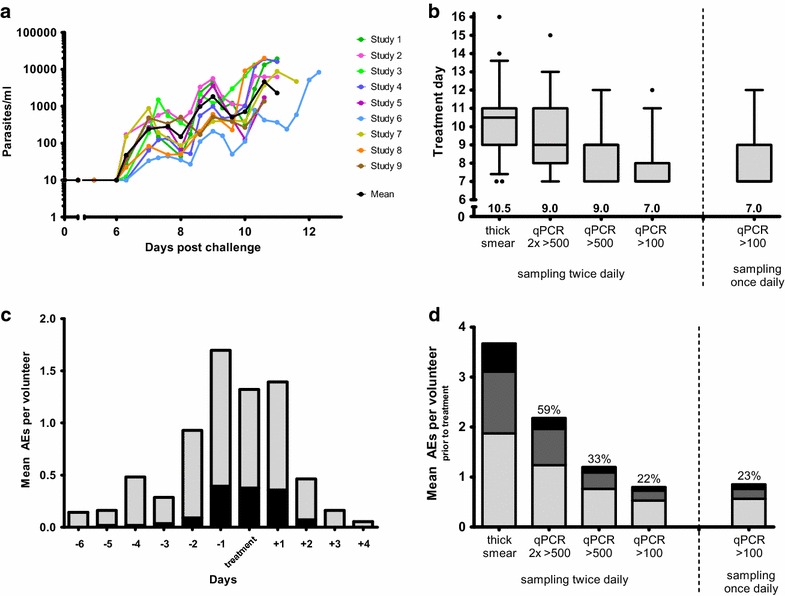
Parasitaemia at different thresholds of qPCR and association with adverse events. **a** Mean parasitaemia by qPCR from a total of 55 malaria naïve volunteers undergoing CHMI by five NF54 infected mosquito bites in nine trials. **b** Day of positive thick smear or positive qPCR at different parasite density thresholds as starting day of curative treatment. *Box*-and-*whisker plots* show the median, first and third quartiles and 5–95th percentiles. *Numbers* above the *x*-axis are median treatment days. **c** The mean number of adverse events per volunteer occurring prior to and after treatment. *Gray* total adverse events, *Black* grade 3. **d** The mean number of adverse events per volunteer occurring prior to thick smear positivity compared to different parasite thresholds for initiation of treatment. Percentages above the *bars* show the percentage of total AEs that occur relative to thick smear. *Black* grade 3, *dark gray* grade 2, *light gray* grade 1

Fifty-five malaria naïve volunteers in nine trials received a challenge infection with bites from five NF54 infected mosquitoes. Geometric mean parasitaemia curves generated from retrospective qPCR data were similar between trials, Fig. 1a. These volunteers received anti-malarial treatment at positive thick blood smear at a median of 10.5 days post-challenge (range 7.0–16.0). Based on the retrospective qPCR data, initiating treatment based on qPCR can gradually decrease the duration of parasitaemia, depending on the treatment threshold and blood sampling frequency used, Fig. 1b. When two consecutive positive qPCR measurements above 500 parasites/ml are used as a criterion to initiate treatment, volunteers are treated at a median of 9 days post CHMI. When only a single positive qPCR is required to initiate treatment, the mean day of treatment decreases further. Using the threshold of 100 parasites/ml blood, the median duration of parasitaemia would decrease by 3.5 days.

All solicited adverse events that were possibly, probably or definitively related to the CHMI occurring between day 5 post-infection and the end of the study were collected. Fifty-five percent of all adverse events and 39 % of severe adverse events occurred prior to the initiation of anti-malarial treatment (Fig. 1c). Importantly, only 22 % of the total adverse events and 13 % of grade 3 adverse events before treatment occurred before parasitaemia reached 100 parasites/ml (Fig. 1d).

Once daily blood sampling for qPCR (threshold of 100 parasites/ml), instead of twice daily sampling, did not influence the median treatment day, Fig. 1b. Five volunteers (9 %) would have been treated 24 h earlier when sampling for qPCR twice daily. However, the mean number of adverse events before treatment increased only minimally when once daily sampling was used, Fig. 1d.

CPS immunization induces dose-dependent protection against CHMI [12]. Partial protection was determined by time to parasitaemia and mean parasite density of the first wave, as estimation of the liver parasite load [4]. Since both parameters depend on the method of parasite detection and treatment threshold used, it was retrospectively assessed whether the proportion of volunteers with partial protection changed with qPCR sampling once daily and initiation of treatment based on a single qPCR above 100 parasites/ml. Table 2 shows that differences in pre-patent period and mean parasitaemia of the first wave for ten partially protected volunteers and controls [11, 12] gave similar outcomes when using microscopy or qPCR.

**Table 2:** Partial protection after CPS immunization as detected by thick smear or retrospective qPCR

	Number	Pre-patent period (days)			Parasitemia 1st peak (log)		
		Mean	SD	P value	Mean	SD	P value
Positive thick smear^a^							
CPS-immunized (partially protected)^b^	10	12.2	1.85	0.006	1.00	0.56	0.02
Controls (unprotected)	9	9.7	2.05		2.07	1.07	
Positive qPCR^c^							
CPS-immunized (partially protected)^b^	10	9.6	2.06	0.035	1.10	0.67	0.04
Controls (unprotected)	9	7.9	1.83		1.99	1.06	

Differences between mean pre-patent periods were determined by Mann–Whitney U test in ten partially protected and nine control volunteers after CPS immunization [11, 12]. Parasitaemia of the first parasite wave was estimated by determining the geometric mean parasitaemia from 6.5 to 8.0 days after challenge. Differences in the mean parasitaemia of the first peak was determined by an independent samples *t* test

^a^Threshold of 4000 parasites/ml and twice daily blood sampling

^b^Only volunteers with patent parasitemia included in the analyis

^c^Threshold of 100 parasites/ml and once daily blood sampling

A tentative diagnostic replacement of microscopy by qPCR requires a reliable test outcome. A total of 778 retrospective qPCR tests have been performed in 35 fully protected volunteers without a single qPCR above 100 parasites/ml. In the same studies, performed between 2010 and 2012, 107 qPCR standard curves were generated using serial dilutions of blood samples with known parasite densities, diluted from isolated ring stages whose concentration had been determined by microscopy. At densities of 20, 50 and 100 parasites/ml, the parasitaemia in these samples was correctly quantified (less than 5 % deviation between duplo samples) in 63 % (57/107), 87 % (93/107) and 96 % (103/107) of the samples, respectively. With recent introduction of a new standardized reagents mix for the DNA extraction in 2014 (MagNA Pure LC Total Nucleic Acid Isolation Kit, Roche Diagnostics), 81 of 82 standard curve samples with 100 parasites/ml and 79 of 82 samples with 50 parasites/ml were correctly measured. The combined data indicate that qPCR with threshold of 100 parasites/ml can be reliably used for diagnosis in the CHMI model, with a sensitivity of 99 % and a specificity of 100 %.

## Discussion

This retrospective qPCR analysis shows that the duration of blood stage parasitaemia in CHMI volunteers can be shortened by 3.5 days compared to thick blood smear if a treatment threshold of 100 parasites/ml is used. This threshold has a sensitivity of 99 % and a specificity of 100 %.

Shortening the duration of parasitaemia in volunteers after CHMI has several potential advantages. Most importantly, an increase in safety as malaria symptoms are related to the height and duration of parasitaemia, and the potential to greatly decrease the burden for volunteers. Over half the adverse events after CHMI occur prior to thick smear positivity. This analysis shows that anti-malarial treatment of volunteers when parasitaemia reaches 100 parasites/ml will lead to a 78 % reduction in the number of adverse events occurring before treatment. Presumably, treatment of volunteers at lower parasitaemia will also lead to a decrease in adverse events occurring after treatment.

If prospective qPCR diagnostics are introduced with a low threshold (100 parasites/ml), once daily blood sampling will suffice without the need for a second sample within 24 h, as there appears to be only a slight effect on the duration of parasitaemia and/or the number of adverse events. Five volunteers (9 %) would have been treated 24 h earlier when sampling for qPCR twice daily. Notwithstanding, we still favour once daily sampling considering the great burden of twice-daily blood sampling and the absence of a significant increase in the number of AEs at that very low parasitaemia. Shortening the duration of parasitaemia and decreasing the frequency of blood sampling will significantly reduce the follow-up of CHMI volunteers. Given the intensive visit schedule for volunteers, requiring multiple personnel and safety laboratory evaluations, the reduced follow-up period will substantially simplify the conduct of these trials, which will also lower CHMI costs.

However, these benefits should not compromise the scientific value of the trial. This study shows that using these diagnostic criteria will not impede the ability to discriminate the delay in parasitaemia and/or reduction in mean first wave parasitaemia as proxy for parasite liver stage development that occurs when a vaccine provides partial pre-erythrocytic protection. Therefore, using once daily qPCR with 100 parasites/ml threshold will likely provide a similar primary outcome of protective vaccine efficacy in prospective studies. However, the standard deviations of both mean time to parasitaemia and mean first wave parasitaemia in the vaccination groups increased in this analysis. Consequently, when a relatively smaller difference is anticipated between vaccinees and controls, use of these qPCR criteria may require an increase in sample size to obtain sufficient statistical power.

Evaluation of qPCR data from 35 CPS-immunized and protected volunteers shows that since the introduction of the current qPCR method at Radboudumc, LUMC and the Harbour Hospital in 2010, no immunized and fully protected volunteers developed a positive qPCR after challenge above 100 parasites/ml. Using this qPCR method, parasites can be detected at a threshold of 50 parasites/ml with about 96 % sensitivity and at 100 parasites/ml with 99 % sensitivity. Therefore, the test clearly has sufficient accuracy for diagnostic purposes at these centres. A possible hazard of using a single positive qPCR as a criterion to initiate treatment is the risk of false-positives by cross-contamination or accidental sample switching, especially since treatment will now often be initiated in the absence of clinical symptoms. To minimize this risk, it is important to set up quality control steps not only within the qPCR test but in the conduct and logistics of the qPCR as well. Prior to a CHMI study, qPCR standards are generated and validated, and the same standard is used throughout an entire study. In order to ensure comparability of CHMI data between centres it will be a logical next step to standardize the PCR assay, or make commercially available *P. falciparum* qPCR standards.

Andrews et al. [17] first demonstrated the increased sensitivity of qPCR compared to thick smear, and recognized that qPCR could be used to initiate earlier treatment, at a threshold of 1000 parasites/ml [17]. However, recent advances in qPCR methodology, such as the use of an automated system for extraction, has improved sensitivity at low parasite densities. The current analysis shows that this has made it possible to lower the treatment threshold much further. Likewise, other CHMI study centres have also repeatedly shown that qPCR first becomes positive 2–4 days before thick blood smear when both are determined [18–21]. Similarly, studies assessing blood stage drugs or vaccines have already begun to use qPCR as a primary outcome, and have confirmed its sensitivity and specificity [22]. In 2014 Kamau et al. analysed parasitological data from 16 subjects undergoing CHMI in two trials. They also showed that qPCR is positive 2–7 days before thick smear [23]. Based on their analysis, the authors recommend treatment after CHMI after two (not necessarily consecutive) positive qPCRs of which one is above 2000 parasites/ml. This threshold was chosen to assess parasite multiplication rates requiring at least two replication cycles. For evaluation of pre-erythrocytic vaccines, however, a treatment threshold of 100 parasites/ml will be sufficiently adequate. This analysis shows that different qPCR thresholds can be chosen to assess the duration of parasitaemia. For example, using two consecutive positive qPCRs above 500 parasites/ml as a threshold, prolongs the median pre-patent period to 9 days. Different qPCR treatment thresholds will therefore lead to different durations of parasitaemia. In this way, CHMI can be made a fit-for-purpose model matching the diagnostic qPCR protocol with the considered primary endpoints.

Although retrospective analyses should be interpreted prudently in general, the predictive value of this study can likely be met with confidence since retrospective qPCR data have been remarkably consistent over time between CHMI trials, and CHMI centres [3]. Therefore, PCR may be preferred for diagnosis and treatment when evaluating the protective efficacy of pre-erythrocytic vaccines [19].

## Conclusions

After CHMI, qPCR becomes positive on average 3.5 days before thick blood smear. This analysis shows that depending on the threshold used, treatment based on qPCR diagnostics can greatly reduce the pre-patent period and the number of AEs occurring before treatment. Furthermore, these data demonstrate for the first time that qPCR has sufficient sensitivity and specificity to use 100 parasite/ml as a treatment threshold without affecting trial outcome related to infection and pre-erythrocytic protective efficacy. Therefore, the implementation of these diagnostics would improve safety while reducing the clinical burden and costs without compromising the evaluation of protective efficacy.

## Abbreviations

CHMI: controlled human malaria infection
CPS: chemoprophylaxis and sporozoite immunization
qPCR: quantitative polymerase chain reaction
CMO: Radboudumc Committee on Research Involving Human Subjects
CCMO: Central Committee on Research Involving Human Subjects of The Netherlands
AEs: adverse events
