# Erratum to: Diagnosis and treatment based on quantitative PCR after controlled human malaria infection

**DOI:** 10.1186/s12936-016-1571-4

**Published:** 2016-10-28

**Authors:** Jona Walk, Remko Schats, Marijke C. C. Langenberg, Isaie J. Reuling, Karina Teelen, Meta Roestenberg, Cornelus C. Hermsen, Leo G. Visser, Robert W. Sauerwein

**Affiliations:** 1 Department of Medical Microbiology and Radboud Center for Infectious Diseases, Radboud University Medical Center, PO Box 9101, 6500 HB Nijmegen, The Netherlands; 2 Department of Infectious Diseases, Leiden University Medical Center, PO Box 9600, 2300 RC Leiden, The Netherlands

## Erratum to: Malar J (2016) 15:398 DOI 10.1186/s12936-016-1434-z

After publication of the original article [1], it came to the authors’ attention that there were errors in the affiliations, and in the Acknowledgements section.

Firstly, the affiliation of Meta Roestenberg was originally given as 1—Department of Medical Microbiology and Radboud Center for Infectious Diseases, Radboud University Medical Center. In fact Meta Roestenberg is affiliated to 2—Department of Infectious Diseases, Leiden University Medical Center.

Secondly, the name of a department and two individuals should have been added to the Acknowledgements section. Eric Brienen, Lisette van Lieshout and the Department of Parasitology of the LUMC should have been included. The Acknowledgements section therefore read as follows:

‘We would like to thank Geert-Jan van Gemert, Marga van de Vegte-Bolmer, Rianne Siebelink-Stoter, Wouter Graumans and Theo Arens for their support of the controlled human malaria infection studies at the Radboud University Medical Center. We thank all the clinical staff who performed the controlled human malaria infection trials, Matthew McCall, An-Emmie Nieman, Else Bijker, Guido Bastiaens, Maurits van Meer, Linda Wammes, Andre van der Ven, Quirijn de Mast, Perry van Genderen, Jaap van Hellemond, Jorien Wiersma, Eric Brienen, Lisette van Lieshout and the entire staff of the Clinical Research Center Nijmegen and the Departments of Medical Microbiology and Parasitology of the LUMC. We thank Anja Scholzen for her advice and input in the data analysis.’
